# Guided Self-Help Behavioral Activation Intervention for Geriatric Depression: Protocol for Pilot Randomized Controlled Trial

**DOI:** 10.2196/18259

**Published:** 2020-09-24

**Authors:** Xiaoxia Wang, Xiaoyan Zhou, Hui Yang

**Affiliations:** 1 Department of Basic Psychology College of Psychology Army Medical University Chongqing China; 2 Department of Clinical Psychology Mental Health Center of Chongqing Chongqing China

**Keywords:** psychiatry, clinical, psychology, geriatric depression, guided self-help, behavior activation, behavior inhibition, behavior treatment

## Abstract

**Background:**

Aging is a social concern. The increased incidence of depression in older populations in China poses a challenge to the health care system. Older adults who are depressed often suffer from a lack of motivation. Behavioral activation treatment, an evidence-based guided self-help treatment, is effective in reducing anhedonia and amotivation in depression; however, the efficacy of guided self-help behavioral activation in older adults with depression is not yet known.

**Objective:**

The aim of this study is to pilot a self-help guided intervention for the treatment of depression in older adults.

**Methods:**

This study has been designed as a pilot randomized controlled trial with inpatients (n=60; to be randomly allocated 1:1) between the ages of 60 and 70 and who have major depressive disorder. Patients attending clinical psychological clinics at the Mental Health Center of Chongqing will be randomized to either receive guided self-help behavioral activation (intervention) or to be on a 6-week waiting list (control). Participants in the treatment group will receive 6 sessions of guided self-help behavioral activation delivered over the telephone. The waiting list control group will receive the intervention after a period of 6 weeks. Exclusion criteria will be individuals who are at significant risk of harming themselves or others, who have a primary mental health disorder other than depression, or who have an intellectual disability that would hamper their ability to participate in the intervention. Effects of the treatment will be observed using outcomes in 3 domains: (1) clinical outcomes (symptom severity, recovery rate), (2) process variables (patient satisfaction, attendance, dropout), and (3) economic outcomes (cost and resource use). We will also examine mediators of outcomes in terms of patient variables (behavioral activation or inhibition motivation). We hypothesize that guided self-help behavioral activation will have a beneficial effect.

**Results:**

The study was approved by the research ethics committee of the Mental Health Center of Chongqing in November 2019. As of July 2020, recruitment had not yet begun. Data collection is expected to be completed by December 2020. Data analysis is expected to be completed by June 2021. Results will then be disseminated to patients, to the public, to clinicians, and to researchers through publications in journals and presentations at conferences.

**Conclusions:**

This will be the first study in China to investigate guided self-help interventions for patients who are older adults and who are depressed, a group which is currently underrepresented in mental health research. The intervention is modular and adapted from an empirically supported behavioral activation treatment for depression. The generalizability and broad inclusion criteria are strengths.

**Trial Registration:**

Chinese Clinical Trial Register ChiCTR1900026066; http://www.chictr.org.cn/showprojen.aspx?proj=43548

**International Registered Report Identifier (IRRID):**

PRR1-10.2196/18259

## Introduction

With the increase in life expectancy and low fertility rate, the health of the aging population has become a social concern. The World Health Organization defines older adults as individuals over 65 years of age (or over 60 years of age, in some instances). Chongqing is among the provinces of China that have a rapidly aging population. According to the data from China’s 2010 population census, the percentage of the population over 65 years of age (14.51%) is higher in Chongqing than those in other provinces in China. In 2020, the population that is 65 years and older in Chongqing is predicted to grow to approximately 3.93 million [1].

With the increase in the aging population, depression in older adults has become the most important public health issue and can significantly increase the total cost of medical services [2]. Geriatric depression specifically refers to primary depression that first occurs after the age of 60 and which cannot be explained by physical symptoms or other organic diseases [3]. In a 2006 nationwide survey, among urban and rural older adults who were assessed with 15-item Geriatric Depression Scale, 13.6% and 25.5% demonstrated moderate or severe depressive symptoms, respectively [4]. Specifically, the aggregate prevalence of depression in the central and western regions (33.7%) was significantly higher than that in the eastern regions (19.1%) of China (measured with Geriatric Depression Scale or Center for Epidemiologic Studies Depression scale) [5]. Among older adults in Chongqing, 24.3% had depressive symptoms (a score greater than 11 on the 30-item Geriatric Depression Scale) [6]. People with chronic diseases, with physical disabilities, and who were living alone, as well as women and older adults in rural areas were more likely to suffer from depression [7].

Evidence-based treatment of geriatric depression mostly focuses on pharmacological treatment [8]. Recent reviews support the efficacy of psychosocial interventions in the acute treatment of geriatric depression [9-11]. A systematic review of randomized controlled trials revealed that behavioral therapy in older adults with depression had comparable effectiveness to those of alternative psychotherapies, such as cognitive therapy or brief psychodynamic therapy [12], as well as to that of antidepressant medication [13]. Behavioral activation is a standalone behavioral therapy developed for the treatment of depression which aims to increase positive reinforcement (such as obtaining a sense of pleasure and control) after the patient engages in antidepressant behavior (such as completion of scheduled activity or active social engagement), which then improves depression symptoms [14]. The monitoring and scheduling of activities are common components of behavioral activation treatment [15]. Activity scheduling, as the effective component of behavioral activation, when compared with controls showed significant effect size [16].

The brevity and simplicity of training and supervision, as well as multiple delivery modes (individual or group, face-to-face, or mobile-based) of behavioral activation may allow greater access, including in primary care and home settings. Currently, depression in older adults is treated mainly in primary care or home settings [17] and is often inadequately treated [8]. As mental health telehealth service matures, self-help treatment can be performed regularly under the guidance of therapists by using mobile technology such as smartphones and the internet. Compared to drug treatments, patients are more inclined to receive guided self-help therapy and psychotherapy [18]. Furthermore, according to the National Institute for Health and Care Excellence guidelines [19], guided self-help interventions delivered without human support or guidance have supporting evidence (degree II), and developing positive activities (the key component of behavioral activation) have supporting evidence (degree I) [20]. Moreover, self-administered behavioral activation interventions based on a self-help book [21] have significantly decreased mild or subthreshold depressive symptoms in late-life individuals (age range 65 to 89) [22]; however, there has still been no report on the implementation of guided self-help behavioral activation interventions for inpatients with geriatric depression. The inpatient group was selected because medical staff have received training to provide professional and systematic psychotherapy (such as cognitive behavior therapy or psychodynamic therapy) and drug intervention, which would not be accessible in the community.

## Methods

### Overview

A guided self-help behavioral activation intervention will be used to implement psychological education about depression and treatment plans for patients with geriatric depression; the behavioral activation intervention app developed by our research group will be used for monitoring and scheduling pleasurable activities. The Geriatric Depression Scale combined with the Behavioral Activation and Behavioral Inhibition scale will be used to investigate the efficacy of behavioral activation on inpatients with geriatric depression.

### Participants

A total of 60 older adults who meet the diagnostic criteria of geriatric depression will be recruited. Participants will be randomly assigned to either the intervention group or the control group, with 30 individuals in each group.

Inclusion criteria are patients conforming to the ICD-10 diagnostic criteria (F32: Major depressive disorder, single episode); residing in Chongqing for more than 6 months; who can communicate barrier-free and are able to complete questionnaires independently or with assistance; who are aged from 60 to 70 years, with an education level of junior high school or above; and who frequently use smartphones (to be able to receive and check text messages and to be able to perform psychological assessments using a simple visual scale).

Exclusion criteria are patients with severe physical illness and organic brain diseases; with confirmed schizophrenia spectrum, psychoactive substance use, or significant neurodevelopmental disorders; with bipolar disorder; with a recent history of severe infection and fever; with severe suicidal tendencies; or with other conditions deemed as not suitable for inclusion after evaluation by researchers.

### Study Design

**Figure 1:**
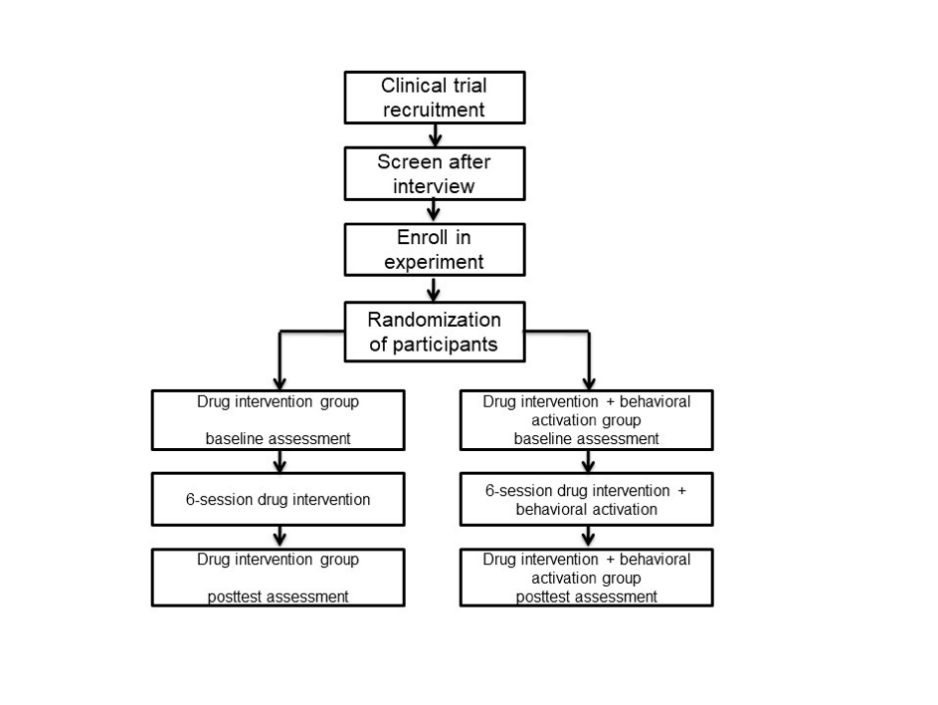
Study design of guided self-help behavior activation intervention for geriatric depression.

The behavioral activation group and the control group will both receive selective serotonin reuptake inhibitor treatment, conventional care, and health education; however, behavioral activation therapy will only be used in the intervention group, will be in accordance with the behavioral activation treatment for depression manual [23], and will be performed for 40 to 50 minutes twice each week for 3 weeks (Figure 1). During the intervention, the therapist provides education and behavioral skills for geriatric depression. Additionally, the behavioral intervention software that we developed will be used to assess changes in depression symptoms, to assess psychological mediators, and to monitor the completion of behavioral activation tasks.

### Treatment Sessions

The intervention is designed as 6 treatment sessions. The first session provides psychological education about depression. The treatment concept and the status and importance of daily monitoring in the treatment process are introduced to participants. The second session involves a review of the previous treatment course to introduce the value and concepts of the activity plan. In different life domains (such as family relationships, learning and work, leisure activities, and hobbies and interests), participants and therapists are inspired through suggestion and writing tasks to list a series of activities that they feel are valuable and enjoyable. The activity schedule is listed together with therapists to allow participants to obtain maximum positive reinforcement in life. During the third to fifth sessions, the treatment concept is constantly reviewed and revised. Participants are asked to make an activity to-do-list using the activity schedule. The sixth session is used to discuss the completion of the treatment, evaluate treatment progress, and teach participants to prevent recurrence using the knowledge and skills learned from treatment [24] (Table 1). After each intervention, the behavioral activation intervention software that we developed assists individual self-learning (psychological knowledge of depression and behavioral activation), establishment of an activity schedule, and monitoring of daily emotional changes. Each session uses the self-rating scale to monitor depression symptoms and motivation changes in patients.

**Table 1:** Establishment of the treatment courses of behavioral activation therapy.

Course	Content	Tasks
1	1. Understanding basic knowledge of depression 2. Understanding treatment principles 3. Mastering principles and methods of the daily activity monitoring table	1. Finishing the daily activity monitoring table
2	1. Reviewing treatment principles 2. Reviewing the daily activity monitoring table 3. Discussing and solving issues in treatment 4. Discussing life domains and activities that are valuable to visitors	1. Finishing the daily activity monitoring table 2. Finishing life domain value assessment and the activity list
3	1. Reviewing the daily activity monitoring table 2. Reviewing the life domain value assessment and the activity list 3. Choosing and sorting activities that need to be reinforced	1. Finishing the daily activity monitoring table 2. Reviewing and improving life domain value assessment and the activity list 3. Reviewing and improving the activity sorting table
4	1. Reviewing the daily activity monitoring table 2. Reviewing the activity list 3. Signing the behavior agreement 4. Planning daily activities that need to be finished next week	1. Finishing the daily activity monitoring table 2. Supplementing and improving the behavior agreement
5	1. Reviewing the daily activity monitoring table 2. Reviewing the behavior agreement 3. Planning daily activities that need to be finished next week	1. Finishing the daily activity monitoring table 2. Supplementing and improving the behavior agreement
6	1. Reviewing the daily activity monitoring table 2. Planning daily activities that need to be finished next week 3. Preparing to finish up	1. Finishing the daily activity monitoring table 2. Supplementing and improving the behavior agreement

### Quality Control

Study participants will be selected strictly according to the inclusion and exclusion criteria. Medical care staff with patience, technique, and strong responsibility will be chosen for performing guidance. They will receive uniform, rigorous training before performing guidance. Attention will be paid to communication skills and to the protection of patient privacy. The data input and review system will be established to ensure the accuracy of data input and analysis.

### Health Outcomes

The Geriatric Depression Scale [25] is a self-rating depression scale specifically developed for older adults. This instrument includes a total of 30 items. The statistical indicator of this scale is the total score. A total score of 0-10 points indicates no depression symptoms, 11-20 points indicates mild depression symptoms, 21-25 points indicates moderate depression symptoms, and 26-30 points indicates severe depression symptoms. The Behavioral Inhibition System and Behavioral Activation System scale [26,27] has 20 items and is divided into 2 systems: the behavioral inhibition system and the behavioral activation system (including 3 dimensions: reward response, drive, and fun seeking). Each item is assessed using a 4-point Likert scale ranging from “completely agree” to “completely disagree.” The Cronbach α (for all dimensions) ranges from 0.66-0.76 [27]. The reliability after 2 months is approximately 0.59-0.69 [27].

### Efficacy Outcomes

After 6 months, at follow-up, the recurrence rate will be evaluated using the Clinical Global Impression scale [28]. The Treatment Emergent Symptom Scale [29] will also be used to evaluate side effects.

### Statistical Analysis

This study will focuses on clinical outcomes in terms of reduction of symptom severity and on motivation (behavioral activation or inhibition) as mediators, as well as descriptive statistics of recovery rates. The process variables, including patient satisfaction, attendance, dropouts, will be reported. The cost-effectiveness of the intervention will be reported in terms of cost and resource use.

Independent and paired *t* tests will be performed using SPSS software (version 22.0; IBM Corp) to investigate the improvement effect in depression symptoms from the behavioral activation intervention. Amos software (version 17.0; IBM Corp), combined with the mediation effect model, will be used to investigate the mediating effect of the behavioral activation inhibition level on the improvement of depression symptoms from the behavioral activation intervention. The mediation effect model and the path analysis statistical method will be used to investigate the mediating mechanism using the behavioral activation or inhibition motivation level as the treatment effect of the guided self-help behavioral activation intervention.

### Ethics and Dissemination

#### Ethical Approval

This study has been reviewed and approved by the research ethics committee of the Mental Health Center of Chongqing. All participants will be asked to give verbal and written informed consent before inclusion and randomization. The study has been registered with the Chinese Clinical Trial Register (ChiCTR1900026066).

#### Informed Consent

All personal information collected during the study will be kept confidential and will be protected in accordance with the law. Participant names and identities will not be disclosed. The study data will be kept confidential and will be stored at the hospital. Only the medical staff, the ethics committee, and national health administration personnel involved in the study will have access to the confidential information for the necessary supervision and inspection.

#### Ethical and Safety Considerations

The study will obtain basic and psychological information through questions and questionnaires, with no risk of physical damage to the participants. To prevent the possibility that the assessment and intervention may cause the participants to be distressed, a well-trained clinical psychiatrist with over 10 years of professional experience will be prepared to intervene if participants report being distressed.

## Results

As of July 2020, participant recruitment had not yet begun. Data collection is expected to be completed by December 2020. Data analysis is expected to be completed by June 2021. Results will then be disseminated to patients, to the public, to clinicians, and to researchers through publications in journals and presentations at conferences.

## Discussion

This will be the first study in China to investigate guided self-help behavioral activation interventions for geriatric depression—a mental health condition which is currently underrepresented in research. The intervention is modular and adapted from manualized behavioral activation treatment for depression [23]. Patients who are older adults generally exhibit more avoidance behavior, which limits their participation in reinforcing activities (eg, leading to pleasurable or healthy outcome) [30].

The primary study will focus on the efficacy (including symptom reduction and remission rate) of the blended intervention (behavioral activation plus drug intervention) relative to that of the drug intervention. The conclusions of the study will be restricted to inpatients who are older adults with depression and who have no severe comorbid physical illness and organic brain diseases. Behavioral activation has great potential to relieve cognitive burden in patients, which may be most apparent in patients who are older adults with depression and with limited education. Furthermore, behavioral activation is easier to learn and to administer than other psychological interventions are and may therefore be more cost-effective for medical service providers. This study will provide data that will allow the effectiveness of behavioral activation to be evaluated in real-world participants with comorbid medical conditions and in community settings in future studies.

## Abbreviations

ICD-10: International Classification of Diseases, 10th edition
